# Phagocytosis in the Brain: Homeostasis and Disease

**DOI:** 10.3389/fimmu.2019.00790

**Published:** 2019-04-16

**Authors:** Dylan A. Galloway, Alexandra E. M. Phillips, David R. J. Owen, Craig S. Moore

**Affiliations:** ^1^Division of BioMedical Sciences, Faculty of Medicine, Memorial University of Newfoundland, St. John's, NL, Canada; ^2^Division of Brain Sciences, Department of Medicine Hammersmith Hospital, Imperial College London, London, United Kingdom

**Keywords:** phagocytosis, microglia, macrophage, neurodegeneration, neuroinflammation

## Abstract

Microglia are resident macrophages of the central nervous system and significantly contribute to overall brain function by participating in phagocytosis during development, homeostasis, and diseased states. Phagocytosis is a highly complex process that is specialized for the uptake and removal of opsonized and non-opsonized targets, such as pathogens, apoptotic cells, and cellular debris. While the role of phagocytosis in mediating classical innate and adaptive immune responses has been known for decades, it is now appreciated that phagocytosis is also critical throughout early neural development, homeostasis, and initiating repair mechanisms. As such, modulating phagocytic processes has provided unexplored avenues with the intent of developing novel therapeutics that promote repair and regeneration in the CNS. Here, we review the functional consequences that phagocytosis plays in both the healthy and diseased CNS, and summarize how phagocytosis contributes to overall pathophysiological mechanisms involved in brain injury and repair.

## Introduction

Phagocytosis is the process through which cells recognize, engulf, and digest large particles (>0.5 microns), including, but not limited to, bacteria, apoptotic cells, and cell debris. Phagocytosis is a receptor-mediated process involving three major steps: “find me,” “eat me,” and “digest me,” with each of these steps being regulated by multiple receptors, unique molecules, and signaling pathways. Specific receptors involved in phagocytosis can be either opsonic (i.e., Fc receptors, complement receptors) or non-opsonic (i.e., C-type lectin receptors, phosphatidylserine receptors). Following recognition by phagocytic receptors, the plasma membrane extends around the phagocytic target in an actin-dependent manner, with particles ultimately being enclosed within a vesicular phagosome. Following formation, this nascent phagosome proceeds through a series of maturation steps, culminating in fusion with lysosomes (phagolysosome) for the eventual destruction of the phagocytosed particles. Importantly, following destruction, byproducts must be effectively dealt with by the phagocytic cell, either through storage, recycling or efflux mechanisms. The basic cell biology of phagocytosis has been extensively reviewed elsewhere (1).

Adding additional complexity to phagocytosis is the requirement for specific outcomes in the context of different phagocytic targets. For example, while recognition and phagocytosis of bacteria requires rapid induction of proinflammatory responses, a similar reaction to apoptotic cells induces detrimental autoinflammation (2). As such, specific immune responses to phagocytic targets are tailored to by a variety of context-dependent signals, including the engagement of phagocytic receptors that utilize distinct inflammatory signaling pathways (pro vs. anti-inflammatory) and microenvironment-derived signals that promote quiescence or inflammation (3, 4).

**Figure 1:**
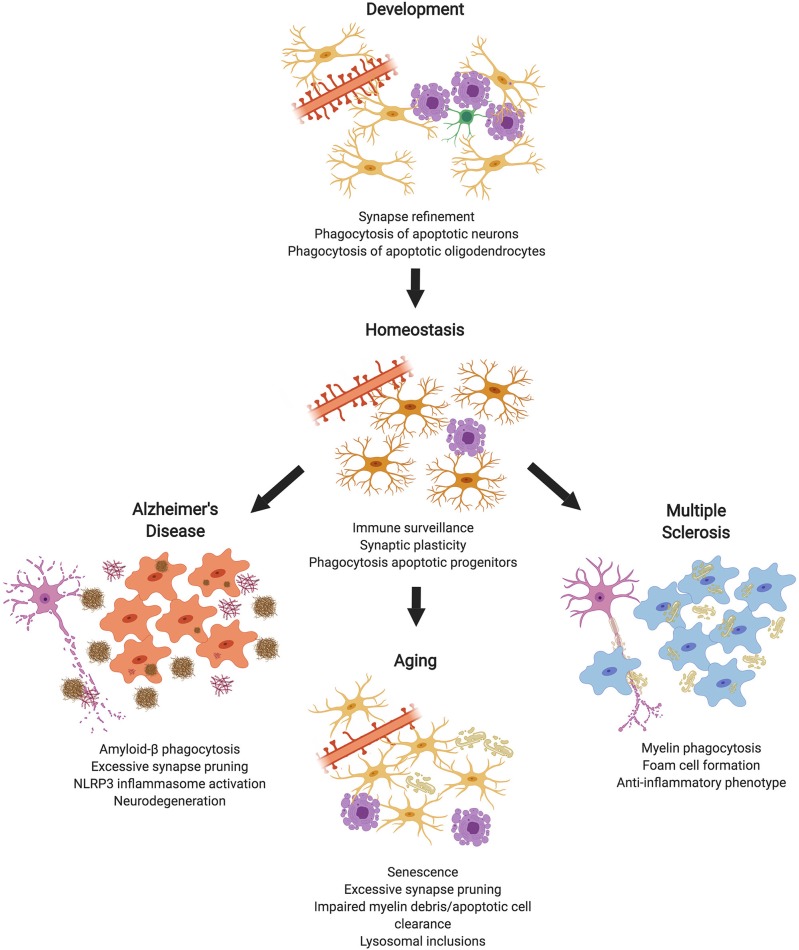
Microglial Phagocytosis in the CNS. During development, microglial phagocytosis is essential for the refinement of excessive synapses, as well as the removal of apoptotic neurons and oligodendrocytes that are overproduced during development. Homeostatic microglia in the adult brain constantly survey the brain's parenchyma, contributing to synaptic plasticity and phagocytosing apoptotic progenitor cells. With advanced age, microglia undergo senescence, display impaired debris clearance, and excessively prune synapses. In diseases, such as Alzheimer's or multiple sclerosis, microglia act as key contributors to pathology, which is partially mediated by phagocytosis of substrates, such as amyloid-β or myelin debris (made in ©BioRender - biorender.com).

Within the CNS, phagocytosis is a critical process required for proper neural circuit development and maintaining homeostasis. To assist in maintaining homeostasis in the CNS, synapses, apoptotic cells, and debris must be continuously removed to maintain optimal neural function. While phagocytosis is primarily attributed to microglia (the professional phagocytes in the CNS), non-professional phagocytes (e.g., astrocytes or oligodendrocytes) may also participate (5). Arising from discrete pathologies, specific phagocytic targets, such as insoluble protein aggregates or myelin debris add further burden to the phagocytic machinery within the CNS. It has been hypothesized that failures during phagocytic processes may actually promote inflammation and/or neurodegenerative processes. Herein, we review how phagocytosis contributes to both the maintenance of homeostasis and disease within the CNS (Figure 1).

## Phagocytosis in CNS Development and Homeostasis

### Synapse Elimination

Within the developing CNS, phagocytosis is necessary for the refinement of synaptic connectivity, as the developing CNS overproduces both neurons and synapses (6). Furthermore, the removal of unwanted synapses refines neural networks, thus contributing to learning and memory (7). Microglia are important cells that execute the pruning of synaptic connections, utilizing immune signaling pathways, such as the complement pathway, although other signaling pathways also contribute (8). Microglia continuously survey the brain's parenchyma (9) and make frequent physical contacts with synapses that are mediated in part by sensing neuronal activity via nucleotides, such as ATP or ADP, and suggest that nucleotides may act as “find me” signals that guide microglial processes toward active synapses (10, 11). Studies investigating synapse pruning have relied extensively on the developing visual system, which is a well-defined sensory system that allows for easy manipulation. Modulation of neuronal activity within the visual system using visual deprivation (to reduce the frequency of action potentials) has demonstrated that neuronal activity is essential for synaptic pruning by microglia (12). Consistent with the role of nucleotides serving as a “find me” signal within the CNS, mice lacking the ADP receptor P2Y12, or pharmacological blockade of P2Y12 signaling, result in impaired synapse pruning within the developing visual cortex (13). Additionally, CX3CR1 knockout mice demonstrate increased hippocampal spine density during development (14), suggesting CX3CL1 may also act as a “find me” signal regulating synapse pruning. However, CX3CL1 is dispensable for synapse pruning within the developing visual system (15). In regards to recognition and engulfment of synapses, the classical complement system has been extensively studied. Briefly, the classical complement system functions via tagging of targets with C1q, which catalyzes the production of C3 convertase. C3 convertase subsequently cleaves C3 producing both C3a (a pro-inflammatory mediator) and C3b, an opsonin that triggers phagocytosis via complement receptors on phagocytes. Early reports demonstrated that the classical complement components C1q and C3 tag synapses for phagocytosis and are required for proper refinement of the developing lateral geniculate (16). Additional reports observed that microglial complement receptor 3 (CR3) is required for the clearance of complement tagged synapses (17). Highlighting the relevance of complement in synapse pruning during development, mice lacking C1q exhibit spontaneous seizure activity as a result of impaired synapse removal (18). The importance of C1q in tagging synapse (19) for elimination is supported by the finding of local apoptotic mechanisms within presynaptic elements that results in C1q accumulation (20). Recent work leveraging the power of correlative light and electron microscopy (CLEM) and live-cell imaging demonstrated that microglia frequently contact synapses within the healthy brain and can be seen engulfing presynaptic, but not post-synaptic, elements via a specialized form of phagocytosis termed trogocytosis, which results in the partial removal of cell constituents (21). While supporting the notion that microglia remove synaptic elements, this study fails to demonstrate that microglia actively phagocytose entire synapses, and further, the authors observed no role for the receptor CR3 in this process. In addition to the classical complement system, both TREM2 and CD47-SIRPα signaling contribute to synapse pruning by microglia (22, 23), although these pathways have been less extensively studied.

In addition to microglia, astrocytes also participate in refining synaptic connectivity (24, 25). It was initially reported that astrocytes participate in the phagocytosis of synapses utilizing the receptors MerTK and MEGF10, whereby astrocyte-specific deletion of these receptors results in a failure to refine synapses in the developing visual system (26). Furthermore, the ability of human astrocytes to phagocytose synapses in dissociated cultures and cerebral organoids has been demonstrated (27, 28), suggesting astrocytes actively prune synapses in the human CNS. Additionally, the Alzheimer-associated gene ApoE regulates the phagocytic capacity of astrocytes and C1q accumulation on synapses during aging (29). It has also been reported that sleep deprivation causes increased synaptic pruning by astrocytes, likely mediated by MerTK (30).

### Removal of Apoptotic Cells

Apoptotic cells are constantly generated and phagocytosed throughout the nervous system during both development and homeostasis. Within the subgranular zone (SGZ) and subventricular zone (SVZ), the major regions containing neural progenitor cells (NPCs), microglia are required to constantly phagocytose apoptotic NPCs throughout the lifespan of the organism. Despite the majority of newborn cells in neurogenic niches undergoing apoptosis, identification of apoptotic cells is difficult due to their rapid clearance by microglia. During inflammatory insult, increased apoptosis of NPCs is coupled to increased phagocytosis by SGZ microglia, suggesting that microglia continue to remove apoptotic progenitors regardless of inflammatory status (31). Mechanistically, the phagocytosis of apoptotic NPCs appears to depend on the TAM family receptors MerTK and AXL, as evidenced by a buildup of apoptotic within the SVZ when these receptors are genetically deleted (32). Interestingly, NPCs may also participate in the phagocytosis of neighboring apoptotic cells and may be required for efficient neurogenesis (33). Apoptotic neurons and oligodendrocytes are also generated throughout development, and phagocytosis is required to clear these cells (34–36). The phagocytosis of apoptotic neurons depends on receptors including TREM2, CD11b, BAI1 and TIM-4, as well as the v-ATPase transporter that is required for the degradation of apoptotic corpses (36–39). Importantly, recent work has identified a novel microglia subset associated with developmental white matter that is specialized for the phagocytosis and removal of apoptotic oligodendrocytes (40). Thus, it appears that microglia may acquire distinct phenotypes that are required for region-specific phagocytic functions which may not be extrapolated from one region to another.

### Phagocytosis During Aging

Within the aging CNS, there is abundant synapse loss and myelin degeneration which is believed to contribute to age-related cognitive decline (41, 42). Microglia- and complement-mediated synapse elimination has been suggested to underlie excessive synapse elimination during normal aging. Increases in complement protein C1q are observed throughout the aged brain, and knockout of C1q prevents age-related cognitive decline, suggesting that excessive synapse pruning during aging potentiates cognitive decline (19). Genetic deletion of the complement component C3 reduces synapse loss and cognitive decline in aged mice, further implicating excessive complement-dependent synapse elimination as a key contributor to age-related cognitive impairment (43). In regards to myelin degeneration, ultrastructural analysis and *in vivo* imaging has demonstrated that large amounts of myelin debris are generated during normal aging, and this debris is continuously phagocytosed by microglia (44, 45). With advanced age, clearance of myelin debris becomes impaired, resulting in insoluble intracellular aggregates (lipofuscin granules) within microglia and microglial senescence (46). This microglial dysfunction is exacerbated by increased production of myelin debris or impairment of lysosomal processing; indicating pathways downstream of engulfment are essential to effectively deal with myelin debris in the healthy CNS. While microglial phagocytosis appears to be an important contributor to the aging brain, further investigations are needed to confirm and expand on these findings.

## Phagocytosis in Disease States

As we begin to understand the roles of microglia in the development and homeostasis of the CNS, it has become increasingly evident that changes in the key functions of these highly active cells can exert significant effects on the progression of multiple CNS diseases (47) (Table 1).

**Table 1:** Current evidence of phagocytosis alterations resulting from variants in disease-associated genes expressed in microglia.

**Gene**	**Associated diseases**	**Models**	**Alterations to phagocytosis**	**References**
TREM2	Alzheimer's disease (48, 49) Frontotemporal dementia (50) Parkinson's disease (50) Nasu-Hakola disease (51)	Primary microglia from TREM2^−/−^ mice	Decreased phagocytosis of Aβ relative to WT microglia	(52)
			Reduced uptake of Aβ-lipoprotein complexes compared with WT and TREM2^+/−^	(53)
			Reduced uptake of *E. coli* particles compared with WT controls	(54)
		Human monocyte-derived macrophages from heterozygous carriers of the TREM2 R62H AD-associated variant	Reduced uptake of Aβ-lipoprotein complexes compared with non-carriers	(53)
		shRNA knockdown of TREM2 expression in primary mouse microglia	Reduced uptake of apoptotic neuronal membranes vs. control shRNA treated cells	(36)
		Immunohistochemical analysis of 5XFAD/TREM2^−/−^ mice	Decreased levels of Aβ within microglial phagosomes vs. WT. Haplodeficient TREM2^+/−^ mice showed no significant reductions in Aβ uptake	(55, 56)
			Increased Aβ load in hippocampus of TREM2 knockout mice	(52)
		Immunohistochemical analysis of APPPS1-21/TREM2^−/−^ mice	Decreased Aβ load in hippocampus of TREM2 knockout mice vs. WT at 2 months	(57)
			Decreased Aβ load in hippocampus of TREM2 knockout mice vs. WT at 4 months	(58)
			Increased Aβ load in hippocampus of TREM2 knockout mice vs. WT at 8 months	
		Immunohistochemical analysis of APPPS1-21/TREM2^+/−^ mice	No difference in Aβ plaque load between WT and TREM2^+/−^ mice at 3 or 7 months old	(59)
		iPSC-derived microglia-like cells from carriers of TREM2 T66M and W50C variants	Decreased uptake of apoptotic neurons by TREM2 variant cells than by controls	(60)
		Non-phagocytic CHO cells transfected with TREM2	TREM2-CHO cells were capable of phagocytosing apoptotic neuronal cells	(61)
CD33	Alzheimer's disease (62, 63)	Primary microglia from CD33^−/−^ mice	Increased uptake of Aβ compared with WT microglia	(64)
		CD33 overexpression in BV2 mouse microglial cell line	Decreased uptake of Aβ compared with control BV2 cells	(64)
		Frontal cortex samples from carriers of protective minor allele SNP rs3865444	Decreased formic acid-soluble Aβ42 levels in carriers of rs3865444 minor (T) allele than in major allele carriers	(64)
TM2D3	Alzheimer's disease (65)	CRISPR-Cas9 knockout in primary human macrophages and U937 human myeloid cell line	Decreased uptake of Aβ and synaptosomes compared with WT	(66)
PU.1	Alzheimer's disease (67)	siRNA knockdown of PU.1 in adult human microglia	Reduced phagocytosis of Aβ compared with controls	(68)
α-Synuclein	Parkinson's disease (69)	Human iPSC-derived macrophages from PD patients carrying SNCA triplication mutations	Increased release of α-synuclein and reduced phagocytosis capability compared with controls	(70)
Progranulin	Frontotemporal dementia (71, 72), Alzheimer's disease (73, 74)	Microglia specific progranulin knockout in AD mice (Grn^flox/flox^/PDAPP_Sw, Ind_ J20)	Decreased microglial phagocytosis of fluorescent beads in acute brain slices and increased hippocampal Aβ plaque-load vs. WT progranulin AD mice	(75)
DAP12	Nasu-Hakola disease (51)	Primary mouse microglia transfected with mutant DAP12 (lack ITAM signaling motif)	Mutant DAP12 microglia phagocytosed less apoptotic neuronal material than control cells	(36)
		Bone marrow-derived macrophages from DAP12^−/−^ mice	Reduced phagocytosis of bacteria	(76)
LRRK2	Parkinson's disease (77, 78)	Microglia and BMDMs from Lrrk2^−/−^ mice	Reduced uptake of latex beads and *E. coli* bioparticles by primary microglia and BMDMs from knockout mice vs. WT.	(79)
			Decreased uptake of beads after injection into midbrain in Lrrk2^−/−^ mice compared with controls	
MerTK	Multiple sclerosis (80, 81)	*in vitro* human microglia and macrophages	Pharmacological blockade of MerTK inhibits myelin phagocytosis *in vitro*	(82)
			MS patient macrophages display reduced expression of MerTK	(83)

### Acute Injury

The cellular response to acute CNS injury, such as traumatic brain injury (TBI), spinal cord injury (SCI), and stroke is multiphasic and has been studied in a range of models. The initial phase involves rapid activation of CNS-resident microglia (9, 84, 85) resulting in pro-inflammatory cytokine release and recruitment of peripheral immune cells, including neutrophils, monocytes, and monocyte-derived macrophages (MDMs), to the lesion site (86, 87). This early response by microglia limits the spread of the lesion (85, 88, 89) but also generates inflammatory cytokines and reactive oxygen species (ROS), which may be detrimental to recovery and contribute to secondary injury if not resolved (90).

Phagocytosis, initially performed by microglia and subsequently by recruited MDMs, acts to restore homeostasis and minimize chronic activation (91). Phagocytosis of apoptotic cells prevents the release of cytotoxic and immunogenic intracellular contents (92, 93) and the removal of damaged myelin has been shown to be important for axon regeneration and remyelination (94–96). Additionally, live neurons may be phagocytosed by microglia during injury (97), inducing a form of cell death termed phagoptosis, which contributes to neuronal cell death during pathological states (98, 99). Importantly, the presence of “don't eat me” signals, primarily CD47-SIRPα or CD200-CD200L signaling, from neurons to microglia suppresses aberrant phagocytosis and is critical for maintaining microglia in a quiescent state [reviewed in (100)].

Recruited MDMs play an important role in post-injury clearance. In a model of cerebral ischemia in mice, MDMs, once localized to the site of injury, have been shown to have a higher phagocytic capacity than microglia (101). However, as demonstrated in a SCI model, recruited MDMs are less capable of processing phagocytic material intracellularly and are also more susceptible to apoptotic and necrotic cell death (102). Therefore, although peripherally-derived MDMs take up more debris post-injury, the inefficient processing of phagocytosed material results in cell stress, which ultimately contributes to the inflammatory milieu.

Whether the activity of infiltrating MDMs following CNS injury is beneficial for repair or a detrimental contributor to inflammation is still disputed (103–106). Following disease onset in models of stroke and SCI, infiltrating MDMs have been found to alter microglial gene expression, downregulating both beneficial (phagocytosis) and neurotoxic (pro-inflammatory) functions (102, 107, 108). Indeed, blockade of MDM infiltration or MDM ablation has been shown to be beneficial in SCI (109–111) and TBI (112, 113). Conversely, other models suggest that preventing the infiltration and functioning of certain MDMs populations in CNS injury results in worsened outcomes (107, 114–116). Contradictory data may be due to inconsistent spatial and temporal assessment of inflammation, phagocytic activity and outcomes in the various models (117) as well as difficulties in differentiating CNS-resident from infiltrating myeloid cells. The use of double transgenic models, such as the Cx3cr1^GFP/+^Ccr2^RFP/+^ mouse, would enable improved distinction between phagocytosis performed by resident microglia and infiltrating monocytes, as used to differentiate these myeloid cell types in studies of stroke (118, 119), SCI (105) and inflammation (120).

### Multiple Sclerosis

Destruction of myelin sheaths within the CNS, as occurs in multiple sclerosis (MS), produces degenerating myelin at sites of injury and inflammation. This degenerating myelin, termed myelin debris, must be cleared from sites of injury to promote timely repair. CNS (but not PNS) myelin acts as a potent inhibitor of oligodendrocyte differentiation (121), and the introduction of exogenous myelin into demyelinated lesions halts oligodendrocyte differentiation at the pre-myelinating stage (122). The removal of myelin debris within MS lesions and experimental animal models, such as experimental autoimmune encephalomyelitis (EAE) or cuprizone-induced demyelination, is primarily mediated by microglia and macrophages. Resident microglia and peripheral macrophages are capable of phagocytosing and degrading large quantities of myelin as highlighted by rapid clearance of myelin debris in animal models, although myelin debris can persist in MS patient lesions (123). Microglia and macrophages differ in their ability to uptake myelin. Specifically, resident microglia demonstrate a greater ability to engulf myelin then peripheral macrophages (124–126), and are more resistant to apoptosis following myelin phagocytosis (102), indicating microglia are more efficient at both engulfing and degrading myelin debris. The mechanism underlying this difference is unknown, although both ontogeny and exposure to the CNS microenvironment likely contribute. For example, astrocyte-conditioned media has been shown to promote myelin phagocytosis by macrophages and microglia *in vitro* (127, 128), suggesting the CNS microenvironment programs myeloid cells for efficient myelin phagocytosis. The phenotype of myeloid cells (pro-inflammatory vs. reparative) also has a large influence on the phagocytic ability of myeloid cells, as inflammatory myeloid cells (e.g., LPS stimulated) display reduced myelin phagocytosis in comparison to reparative, anti-inflammatory myeloid cells (e.g., IL-4 stimulated) (126).

Early investigations into myelin phagocytosis examined the effects of opsonization, demonstrating that both immunoglobulins and complement proteins promote the phagocytosis of myelin, and blocking Fc or complement receptors reduced myelin phagocytosis *in vitro* (125, 129, 130). In addition, evidence from MS lesions suggests that Fc receptors and complement play active roles in myelin phagocytosis (131). Interestingly, myelin debris is capable of activating complement in the absence of myelin reactive antibodies (132). Furthermore, myelin phagocytosis *in vitro* relies on scavenger and C-type lectin receptors for recognition and internalization of myelin debris (125, 133). More recently, the TAM family receptors MerTK and AXL, which bind phosphatidylserine via the bridging molecules Protein S and Gas6, respectively, have been identified as essential regulators of myelin phagocytosis. Within the EAE animal model, deletion of AXL results in increased clinical severity and impaired myelin clearance, while delivery of exogenous Gas6 is protective (134). Loss of AXL/Gas6 during cuprizone-induced demyelination results in increased neuroinflammation and impaired remyelination, indicating that signaling via the apoptotic cell receptor AXL is required to promote the resolution of inflammation following demyelination (135). Studies utilizing human macrophages and microglia have demonstrated that MerTK is an essential phagocytic receptor for myelin, expression of MerTK correlates with myelin phagocytosis *in vitro*, and MerTK levels are reduced within MS patient macrophages (82, 83). Polymorphisms within the MerTK gene are associated with MS susceptibility, suggesting MerTK plays an important role in the development of MS (80, 81). Finally, TREM2 has also been implicated in myelin phagocytosis. TREM2 binds myelin lipids directly to facilitate internalization, and studies using the EAE animal model observed that blockade of TREM2 increases EAE severity, while TREM2 overexpression is protective, in part mediated by effects on clearance of myelin debris (136–138). Moreover, TREM2 KO mice display faulty myelin debris clearance and remyelination in the cuprizone model of toxic demyelination, with TREM2 knockout microglia failing to upregulation genes associated with phagocytosis and lipid metabolism (138, 139).

In addition to myeloid cells, astrocytes have been observed to engulf myelin debris. Within MS lesions, hypertrophic astrocytes contain myelin inclusions (140), and astrocytes have been demonstrated to uptake myelin debris *in vitro* (141). Transcriptomic analysis of astrocytes reveals expression of several complete phagocytic pathways and apoptotic cell receptors, such as MerTK, AXL and LRP1 (142). This is supported by the description of neurotoxic “A1” astrocytes, which downregulate phagocytic receptors including MerTK and show impaired myelin phagocytosis *in vitro* (143). Astrocytes phagocytose myelin at quantities several-fold lower than myeloid cells (144), questioning the functional significance of astrocyte-mediated myelin phagocytosis *in vivo*. Recently, phagocytosis of myelin by astrocytes has been shown to induce the expression of multiple chemokines both *in vitro* and *in vivo* (145), suggesting that astrocytes sense myelin debris and respond by recruiting professional phagocytes to sites of injury. These results are in line with the demonstration that astrocyte ablation impairs myeloid cell recruitment and phagocytosis in the cuprizone model, partly due to lack of CXCL10 expression (146).

### Alzheimer's Disease and CNS Phagocytosis During Aging

Alzheimer's disease (AD) is characterized by the accumulation of extracellular plaques of toxic amyloid-beta (Aβ) and intracellular neurofibrillary tangles. The amyloid cascade hypothesis posits that an imbalance between the production and the clearance of Aβ initiates the pathological cascade of synapse loss, neuron death, and brain atrophy found in AD (147). The contribution of microglia to AD pathogenesis is becoming increasingly recognized as genome wide association studies (GWAS) and transcriptomic analyses highlight links between microglial genes and AD risk, as well as between microglial signaling pathways and disease progression (47).

In the case of the more common, late onset AD (LOAD), it has been argued that impairment in the clearance of Aβ has a greater impact on disease progression than its overproduction (148). As the primary resident phagocytes of the CNS, microglia play an important role in preventing the accumulation of this toxic protein through both phagocytosis and the production of degrading intra- and extracellular enzymes (149). *In vitro*, Aβ initiates cell stress responses, synapse loss, mitochondrial dysfunction, and neuronal apoptosis. In the early stages of AD, microglial function is neuroprotective, acting to clear apoptotic cells and pathological protein aggregates (150) as well as forming a barrier around plaques to restrict their growth and diffusion of synaptotoxic Aβ oligomers (151, 152). However, long term exposure to Aβ induces chronic microglial activation-associated dysfunction known as reactive microgliosis (153), in which phenotypic changes result in the adoption of a continuous pro-inflammatory status and compromised phagocytosis (154–156).

It is important to note that many *in vitro* and *in vivo* phagocytosis assays rely heavily on determining the uptake, but not measuring the subsequent degradation, of pathogenic proteins. In order to prevent intracellular accumulation of Aβ, it must be appropriately degraded and cleared through the endosome-lysosomal pathway (157, 158). The uptake of fibrillar and soluble Aβ has been reported in multiple models of microglia *in vitro* and *in vivo*, however, whether complete degradation of this protein, in particular the fibrillar (f) form, occurs remains disputed. Following *in vitro* culture of control microglia with fAβ, phagosomes have been found to contain incompletely degraded Aβ for up to 20 days (159–161). It has been reported that acidification of microglial lysosomes, for example by treatment with MCSF, can improve the efficiency of intracellular fAβ breakdown (162, 163).

The presence of complement activation in AD pathology has been observed for several decades (164, 165) however, it is only recently that genetic analyses have identified complement components as playing a role in AD pathogenesis (166–169). As discussed previously, complement-mediated pruning of synapses is a key microglial function during development and, whilst complement factors aid plaque clearance (170, 171), phagocytosis of synapses appears to become dysregulated during AD. Synapse loss has been identified early on in AD and correlates strongly with cognitive decline (172, 173). Inhibition or knockout of the complement components C1q, C3, and CR3, required for microglial synapse refinement during development, reduced the synapse loss found in mouse AD models (174–176).

GWAS studies have identified the rare variant R47H in the gene encoding the phagocytic receptor TREM2 as a risk factor for the development of LOAD (48, 49). TREM2 expression is necessary for the phagocytosis of a range of particles (36, 53, 54); TREM2 knockout microglia are less efficient at phagocytosing Aβ than WT microglia (53, 55) and mutations in TREM2 affect the detection of damage-associated lipids by microglia (56), which may explain their reduced ability to take up apoptotic cells (60, 61, 177). It has recently been suggested that TREM2 drives the expression of the scavenger receptor CD36, via the upregulation of C/EBPα (52), augmenting phagocytosis. TREM2 knockout AD models have produced contrasting results regarding amyloid burden (56–59), however it can be argued that this is an indirect measure of microglial phagocytosis of Aβ, as amyloid burden can be altered by other reported TREM2-mediated effects including microglial migration and plaque barrier formation (55, 178, 179).

The ApolipoproteinE–ε4 allele is the greatest genetic risk factor for the development of LOAD (180). APOE has been found to be an endogenous TREM2 ligand (53, 181), suggesting an interaction between the two most significant AD genetic risk factors on the surface of microglia. APOE binds to both Aβ (181, 182) and apoptotic cells (181) and therefore may facilitate the detection and phagocytosis of Aβ and apoptotic cells by TREM2-expressing microglia. The TREM2-APOE signaling pathway has been reported to suppress the homeostatic signature of microglia in several CNS disease models, inducing a shift to a neurodegenerative phenotype (183). Therefore, this signaling axis may exert effects on both beneficial and detrimental microglial functions.

Single-cell RNA-sequencing has identified a new subset of highly phagocytic, AD-associated microglia (DAM) surrounding the plaques in an AD mouse model (184). Interestingly, this subset of microglia was also found in models of aging and ALS so may represent a generalized response to age-related neurodegeneration, or loss of homeostasis, rather than to a specific disease-associated protein. Whether this adoption of a highly phagocytic phenotype is beneficial or deleterious for AD progression has not yet been established.

It has been argued that aging and the expression of genetic risk factors, either independently or in combination, limit the ability of microglia to prevent or slow the pathogenesis of neurodegenerative diseases (185). Age is the single biggest risk factor for the development of LOAD and the recent identification of a unique phenotype of aged human microglia, in which susceptibility genes for both AD and MS were found to be enriched (186), suggests that age could significantly impact microglial function. In the aged brain, microglia exhibit marked changes in their morphology and activity; compared to young cells there is an increase in soma size, a loss of the characteristic tiled tissue distribution, and shorter, less dynamic processes (187–189). Primary mouse microglia demonstrate age-dependent, substrate-specific decreases in phagocytosis of fibrillary Aβ (190) and α-synuclein oligomers (191). Age-related phagocytic activation of microglia, which correlated with cognitive impairment, was reported in aged rhesus monkey brain (192). In this study, immunohistochemical analysis of white matter regions indicated that with age, increasing numbers of microglia simultaneously expressed galectin-3, a phagocytic marker, and HLA-DR MHC II, a marker of microglial activation. However, in another study, the presence of large, heterogeneous intracellular inclusions suggested that increased uptake, but inefficient lysosomal digestion, of particles may be associated with aged microglia (193). Poor Aβ protein degradation is also found in aged mouse microglia (188, 194). Therefore, age-related changes may increase the susceptibility to abnormally folded proteins and accumulating debris, resulting in a loss of homeostasis and the persistence of cytotoxic conditions.

### Other Neurodegenerative Diseases

Microglial phagocytosis has been implicated in a range of other neurodegenerative diseases (195), in particular proteopathic diseases in which the balance of protein production, clearance and degradation becomes dysregulated. In prion disease, the pathogenic form of prion protein (PrPsc) is not taken up by microglia (196), and alters the uptake of other particles (197), resulting in the accumulation of pathology. Multiple studies have demonstrated the ability of α-synuclein (α-SYN), the pathogenic protein found in Lewy bodies in Parkinson's disease (PD), to alter microglial phagocytosis, although this is conformation and expression-level dependent (198). Mutations in LRRK2 are the most commonly found variants in familial PD and recent work has demonstrated that LRRK2 influences myeloid cell phagocytosis via interactions with the actin remodeling protein WAVE2 (79). LRRK2 deficiency in mouse microglia attenuated phagocytosis of beads, whereas expression of PD-associated LRRK2–G2019S augmented phagocytosis in mouse microglia and patient-derived macrophages, which may result in neuronal damage due to overactive phagocytosis during disease (79).

The TREM2 R47H variant associated with AD has also been found to be a risk factor for PD and frontotemporal dementia (FTD), suggesting a common role for TREM2 dysfunction in multiple neurodegenerative diseases (50). Nasu-Hakola disease (NHD) is a progressive, presenile dementia in which phagocytic alterations are a primary cause of pathology. Rare but lethal, NHD is caused by a loss of function of TREM2 or its signaling partner DAP12 (51). Significant demyelination is found in patient brains (199) and in mouse models (200, 201), however signs of Aβ and tau pathology are limited, despite the role of TREM2 in AD (202). Overactive microglial phagocytosis is also a driver of the pathology found in FTD; loss of expression of functional progranulin results in increased C1q production and complement-mediated synapse loss during aging (203).

## Modeling Phagocytosis in the CNS

CNS phagocytosis can be modeled and studied using a range of *in vitro* and *in vivo* techniques. Flow cytometry and microscopy are frequently and easily utilized to assess the uptake by cell lines or primary cells *in vitro* of fluorescently-labeled synthetic or physiological particles, including latex, Aβ, myelin, zymosan, and dextran (204). Flow cytometry allows the rapid assessment of large numbers of cells, whereas microscopy provides additional information on the motility and morphology of the cells as they perform this actin-associated function. Time-lapse microscopy allows the monitoring of the clearance or, in the case of some pathogenic proteins, the persistence of phagocytosed material within the cells. The ability to fluorescently label a range of particles enables disease- or context-specific analysis of phagocytosis, however, in order to ensure the identity of the phagocytic cell, these experiments are frequently performed in monoculture. Monocultures of primary cells are valuable for understanding the morphological changes and cellular pathways of specific substrate uptake during phagocytosis but do not provide information on the interactions between different cell types during this process. It has also been shown that the isolation and culture of microglia rapidly alters their transcriptomic signature (205), so *in vitro* assays may not accurately recapitulate CNS phagocytosis.

*In vivo*, phagocytosis can be inferred in tissue by the expression of phagocytic markers, such as CD206 and CD68, or live cell imaging either transcranially or organotypic cultures. Using acutely prepared and organotypic slices, microglial phagocytosis of apoptotic neurons has been observed (206, 207). These techniques preserve the structure and physiological conditions within the tissue, and, when combined with fluorescent labels, such as lectin, and two-photon or confocal time lapse imaging, allow the study of interactions between different cell types (208). Transgenic mouse lines in which enhanced green fluorescent protein (EGFP) is expressed under the control of myeloid cell-specific gene promoters, including CX_3_CR1 (209), Iba1 (210), and Cfs1r (211), allow live fluorescent imaging of microglial migration and phagocytosis (12).

Species-specific differences between mouse and human cells have been found with respect to phagocytosis (212) so, in the case of investigating human diseases, human studies are necessary. Primary human microglia can be obtained from fetal or *post-mortem* samples, however these resources can be difficult to obtain particularly within acceptable *post-mortem* delay conditions. The recent development of methods for the generation of induced pluripotent stem cell-derived microglia-like cells (iMGLs) allow the study of human microglia function *in vitro* (70, 213–215). Mimicking scarce primary human microglia, iMGLs are capable of phagocytosing synaptosomes, as found during development, and also disease-associated Aβ and tau (213). This model system is valuable for investigating interactions between genetic risk factors and pathogenesis, as recently demonstrated by iMGLs carrying patient-derived TREM2 variants phagocytosing less apoptotic cell material than controls (60), whilst avoiding the caveats associated with mouse models of human disease. It should be noted that these cells have never been exposed to cues arising from the CNS microenvironment, which may alter the differentiation and function of the iMGLs.

Until recently, markers for distinguishing microglia from monocytes were lacking, making it difficult to determine whether phagocytosis was being performed by CNS resident microglia or infiltrating myeloid cells. The discovery of microglia-specific proteins, such as TMEM119 (216), will allow more accurate investigations of the microglial-specific contributions to homeostasis and disease. These tools may also allow the resolution of current discrepancies, including those regarding phagocytosis, found between different disease models (56, 57, 117).

## Conclusions and Perspectives

In this review we have summarized the critical role phagocytosis plays in both CNS homeostasis and disease. While much progress has been made in recent years, many unanswered questions remain. How phagocytosis in the CNS is influenced by numerous factors, such as microenvironment or phagocytic target, have yet to be fully resolved. Additionally, the utilization of novel technologies, including *in vivo* imaging techniques (217), iPSC-derived microglia (213) and high-throughput screens (66), will likely contribute to further identification of phagocytic pathways and consequences of phagocytosis within the CNS. As targeting myeloid cells in neuroinflammatory and neurodegenerative diseases is receiving increased interest (218), drugs modulating phagocytic pathways may emerge as novel therapeutics for brain disease.

## Author Contributions

DG and AP performed the literature search and wrote the manuscript. DO and CM oversaw preparation of the manuscript, and contributed to writing and editing of the manuscript.

### Conflict of Interest Statement

The authors declare that the research was conducted in the absence of any commercial or financial relationships that could be construed as a potential conflict of interest.
