# Corrigendum: Phagocytosis in the Brain: Homeostasis and Disease

**DOI:** 10.3389/fimmu.2019.01575

**Published:** 2019-07-10

**Authors:** Dylan A. Galloway, Alexandra E. M. Phillips, David R. J. Owen, Craig S. Moore

**Affiliations:** ^1^Division of BioMedical Sciences, Faculty of Medicine, Memorial University of Newfoundland, St. John's, NL, Canada; ^2^Division of Brain Sciences, Department of Medicine Hammersmith Hospital, Imperial College London, London, United Kingdom

**Keywords:** phagocytosis, microglia, macrophage, neurodegeneration, neuroinflammation

In the original article, there was a mistake in Figure 1, as well as its legend, as published. It was incorrectly stated that microglia remove “apoptotic oligodendrocyte progenitor cells (OPCs)” instead of “apoptotic oligodendrocytes.” The corrected Figure 1 and its legend appears below.

**Figure 1:**
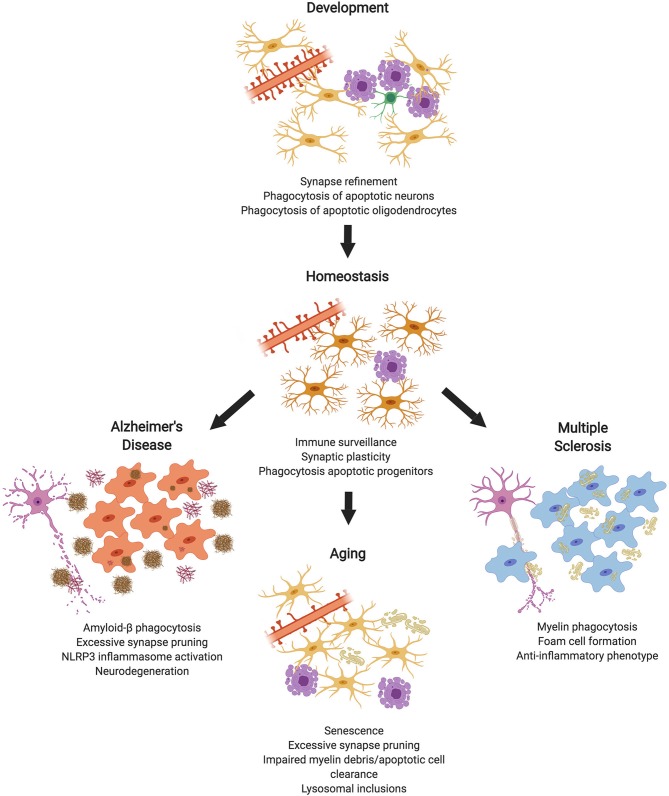
Microglial Phagocytosis in the CNS. During development, microglial phagocytosis is essential for the refinement of excessive synapses, as well as the removal of apoptotic neurons and oligodendrocytes that are overproduced during development. Homeostatic microglia in the adult brain constantly survey the brain's parenchyma, contributing to synaptic plasticity and phagocytosing apoptotic progenitor cells. With advanced age, microglia undergo senescence, display impaired debris clearance, and excessively prune synapses. In diseases, such as Alzheimer's or multiple sclerosis, microglia act as key contributors to pathology, which is partially mediated by phagocytosis of substrates, such as amyloid-β or myelin debris (made in ©BioRender - biorender.com).

The authors apologize for this error and state that this does not change the scientific conclusions of the article in any way. The original article has been updated.

